# Announcement: Vth Conference of the International Society for Trace Element Research in Humans (ISTERH)

**DOI:** 10.1155/MBD.1997.115

**Published:** 1997


					International Center for Trace Element Study
created under the auspices of UNESCO
ISTERH
International Society for Trace Element
Research in Humans
Vth CONFERENCE of the
INTERNATIONAL SOCIETY FOR TRACE ELEMENT
RESEARCH IN HUMANS
(ISTERH)
Lyon, France
September 27 30th 1998
The Fifth International Conference of the ISTERH will be held at "Trace Element-Institute for UNESCO"
in Lyon, France. The Conference will be sponsored by the Institute and by other local and interna-
tional organizations. The programme will be multidisciplinary and will consist of invited papers, work-
shops, oral presentations
and poster sessions. Topics will include the influence of trace elements on gene expression, the
effects of reactive oxygen species on human health and disease, and the newer aspects of essential
and toxic trace elements.
Attention will also be focused on the impact of trace elements in developing countries with special
emphasis on metal toxicity associated with rapid industrial development in the regions of Asia, Africa
and Latin America.
The meeting will be of interest to all scientists involved in trace element research. All accepted ab-
stracts will be published in the official journal of the Society, "The Journal of Trace Elements in Experi-
mental Medicine".
For more information, contact:
Vth CONFERENCE OF THE INTERNATIONAL SOCIETY
FOR TRACE ELEMENT RESEARCH IN HUMANS, September 27 30th 1998
TRACE ELEMENT- INSTITUTE FOR UNESCO
Immeuble Le Condorcet,
1 place de I'Eole
B.P. 7021 F-69342 Lyon Cedex 07, France
Tel. 33 (0) 4 72 80 82 901 Fax 33 (0) 4 78 58 86 71
E-mail: 101711.2322 @ compuserve.corn
115

				

